# Cutaneous non‐tuberculous mycobacterial infections: A retrospective study of 94 cases from Germany

**DOI:** 10.1111/ddg.15910

**Published:** 2025-10-14

**Authors:** Luisa Bopp, Nicolai Deresz, Henning Klapproth, Isabelle Suárez, Jonathan Jantsch, Mario Fabri, Esther von Stebut

**Affiliations:** ^1^ Department of Dermatology and Venereology University of Cologne Faculty of Medicine and University Hospital Cologne Cologne Germany; ^2^ Department of Dermatology University Medical Center Mainz Mainz Germany; ^3^ Department I of Internal Medicine Faculty of Medicine and University Hospital Cologne University of Cologne Cologne Germany; ^4^ Institute for Medical Microbiology Immunology and Hygiene University Hospital Cologne and Faculty of Medicine University of Cologne Cologne Germany; ^5^ Center for Molecular Medicine Cologne (CMMC) Medical Faculty University of Cologne Cologne Germany; ^6^ German Center for Infection Research (DZIF) Partner Site Bonn‐Cologne Cologne Germany; ^7^ Department of Dermatology Jena University Hospital Jena Germany

**Keywords:** fish tank granuloma, Mycobacterium marinum, mycobacteriae, NTM

## Abstract

**Background:**

Cutaneous non‐tuberculous mycobacterial infections (NTM) remain a diagnostic and therapeutic challenge. The aim of this study was to characterize cutaneous NTM infections in Germany over a 24‐year‐period.

**Patients and Methods:**

73 patients with cutaneous NTM infections diagnosed at 17 different German University Hospitals from 2000–2011 and 21 patients treated at the Department of Dermatology of the University Hospital Cologne, from 2010–2024. Retrospective analysis of patient, demographic, clinical information, and data on different diagnostic workups and therapeutic approaches/responses.

**Results:**

Together, 94 cases were analyzed (71% males, mean age 50, > 75% immunocompetent). Exposure to fish tanks was the dominant risk factor. Pathogen detection by nucleic acid amplification test and/or culture was successful in 76%. The most common pathogen was *Mycobacterium (M.) marinum* (> 65%), followed by *M. abscessus/chelonae*. Immunosuppression was associated with NTM other than *M. marinum*. Over 90% were treated with oral antibiotics, in more than 50% with a single antibiotic (mostly clarithromycin). The most common combination was clarithromycin plus rifampicin. The mean duration of therapy was more than 4 months. Complications, adverse events, and relapses were rare.

**Conclusions:**

Our findings highlight that there is a need for standardized diagnostic procedures‐ and therapeutic recommendations for cutaneous NTM infections.

## INTRODUCTION

Non‐tuberculous mycobacteria (NTM) are acid‐fast bacilli ubiquitously present in environmental habitats such as natural waters, soil, and drinking water distribution systems.^1^ Only some of them cause diseases in humans and are facultative pathogens. Manifestation of disease depends on the interaction of the mycobacterium with the host‐immune system. Immunosuppression, for example associated with HIV infection or organ transplantation, as well as biological treatment such as tumor necrosis factor (TNF)‐receptor blockers, increases susceptibility and represents a risk factor for dissemination.^2^, ^3^, ^4^ Pulmonary disease is the most common clinical form of NTM infection, followed by skin and soft tissue infections.^5^

Cutaneous NTM infections are the result of direct inoculation through skin barrier breaks or hematogenous dissemination of a systemic infection.^6^ They can be categorized into two groups: localized superficial (including cellulitis, abscesses, nodules, pustules) and deep infections (subfascial abscesses, tenosynovitis).^7^ The incubation period ranges from 2 to 6 weeks.^8^ Whereas the histopathological spectrum of NTM infections can be variable, granuloma formation is a common pattern.^9^, ^10^ Although the true incidence of cutaneous NTM infections is unknown, an increased incidence in recent years has been suggested, mainly due to increased immunosuppressive therapies, surgical/cosmetic procedures, and improved diagnostics.^11^, ^12^, ^13^, ^14^ Previous trauma (e.g., liposuction) is associated with infections with the rapidly‐growing mycobacteria (RGM) *Mycobacterium (M.) fortuitum*, *M. abscessus*, and *M. chelonae)*, mainly through contaminated water distribution systems.^15^, ^16^, ^17^ Together with *M. marinum*, which is the main cause of infection in fish tank owners and other water‐exposed individuals,^3^, ^18^ they are the most commonly isolated pathogens in cutaneous NTM infections. Diagnosis and initiation of treatment are often delayed due to non‐specific clinical presentation and lack of suspicion for unusual organisms as the source of infection. Moreover, the diagnostics of NTM are complex,^19^ time‐consuming, and pathogen detection is not always successful. There is limited data to guide the management of cutaneous NTM infections. While randomized‐controlled trials are lacking, treatment recommendations are based on expert opinions.^20^ Moreover, common treatment approaches are adapted from the most recent guideline from 2007, which relates primarily to pulmonary disease.^20^ In general, dual or single antimycobacterial therapy, which is administered over several months, sometimes in combination with surgery, is recommended.

This retrospective study was conducted to investigate the clinical and microbial features of cutaneous NTM infections in Germany over a 24‐year‐period. We examined 73 patients diagnosed in 17 different German University Hospitals from 2000–2011 (cohort‐1),^21^ and 21 patients treated in our department in Cologne from 2010‐2024 (cohort–2). In addition to demographic and clinical information, our analysis also includes data on different diagnostic workups and therapeutic approaches and responses. In addition, we re‐evaluated the histopathological slides for cutaneous reaction patterns in samples of cohort‐2.

## PATIENTS AND METHODS

In cohort‐1, we reviewed the medical records of patients with cutaneous NTM infections who presented to 17 different Departments of Dermatology within Germany between 2000 and 2011.^21^ In cohort‐2, data of medical records from patients with cutaneous NTM infections who were treated at the Department of Dermatology of the University Hospital Cologne were reviewed. All cases between 01/01/2010–31/03/2024 were included. Deep infections were not identified. Besides demographic data, predisposing factors, travel information, and symptom duration, the clinical presentation, diagnostic approaches and therapeutic strategies and outcomes were recorded.

We analyzed which diagnostic findings the final diagnosis was based on (NTM detection, histopathology of skin biopsy specimens, cutaneous findings, risk factors [for example fish tank exposure], and response to antimycobacterial therapy). Positive pathogen detection was defined as cases in which NTM was detected by nucleic acid amplification test (NAAT) and/or by culture from a site of infection. Time to diagnosis was defined as the time from symptom onset to diagnosis of infection. Recurrence was defined as evidence of disease at the final visit after cessation of therapy. Lost to follow‐up was defined if there was no documentation of outcome or no final visit. Histopathological slides of cases from cohort‐2 were reviewed by a dermatopathologist to assess epidermal changes, the subtype of granulomatous reaction (suppurative granuloma, non‐caseating tuberculoid, messy granuloma), and the composition, depth and distribution of the (concomitant) immune infiltrate.^22^, ^23^, ^24^

Data from cohort‐1 and cohort‐2 were analyzed separately and – whenever feasible – combined. Qualitative data were summarized by count and percentages, and quantitative data by mean (range) or median (interquartile range, IQR). Chi‐square tests were performed using Prism 10.1.2 software (GraphPad).

## RESULTS

Patient characteristics from both cohorts are found in Table 1. Together, 94 cases were identified (n = 73 in cohort‐1, n = 21 in cohort‐2). Most patients (74% in cohort‐1, 69% in cohort‐2) were male. The mean age was 50 years (range 2–83). The majority of cases (85% in both cohorts) was not travel‐associated. Exposure to fish tanks was the most common risk factor (79.5% in cohort‐1, 33.5% in cohort‐2), with five work‐related expositions in both cohorts together. In cohort‐1, there were significantly more cases with aquatic exposure, especially to fish tanks, while in cohort‐2 there were more cases without specific exposition documented (p = 0.0009).

**TABLE 1:** Patient characteristics.

			Germany (2000–2011)	Cologne (2012–2024)	Combined (2000–2024)
*No. of patients*	*All*		*73*	*21*	*94*
Gender – No. (%)	Female Male Unknown		18→(24.7) 54→(74) 1→(1.3)	8→(38.1) 13→(61.9) 0→(0)	26→(27.7) 67→(71.3) 1→(1.1)
Age at diagnosis	Mean (range) – yr Median (IQR) – yr Unknown – No (%)		50.5→(2–82) 51→(39.5–64.5) 10→(13.7)	48.3→(15–83) 51→(32–58) 0→(0)	50→(2–83) 51→(39–63) 10→(10.1)
Travel history – No. (%)	Yes No Unknown		3→(4.1) 62→(84.9) 8→(11)	3→(14.3) 18→(85.7) 0→(0)	6→(6.4) 80→(85.1) 8→(8.5)
Exposition – No. (%)	Fish tank Pool/pond Preceding liposuction No specific exposition		58→(79.5) 4→(5.5) 0→(0) 11→(15.1)	7→(33.3) 3→(14.3) 1→(4.8) 10→(47.6)	65→(69.1) 7→(7.4) 1→(1.1) 21→(22.3)
Work‐related – No. (%)	Yes No Unknown		3→(4.1) 55→(75.3) 15→(20.6)	2→(9.5) 18→(85.7) 1→(4.8)	5→(5.3) 73→(77.7) 16→(17)
Infected site – No. (%)	Upper Extremity Lower Extremity Trunk/Neck Face Unknown		65→(89) 4→(5.5) 0→(0) 3→(4.1) 1→(1.4)	13→(61.9) 5→(23.8) 2→(9.5) 1→(4.8) 0→(0)	78→(83) 9→(9.6) 2→(2.1) 4→(4.3) 1→(1.1)
Immunosuppression – No. (%)	Yes No	Oral steroid HIV Methotrexate Ciclosporine Rituximab, CLL TNF‐α‐inhibitor Azathioprin	7→(9.6) 3→(42.9) 2→(28.6) 1→(14.25) 1→(14.25) 0→(0) 0→(0) 0→(0) 66→(90.4)	5→(23.8) 0→(0) 0→(0) 0→(0) 0→(0) 1→(4.8) 3→(14.3) 1→(4.8) 16→(76.2)	12→(12.8) 3→(3.2) 2→(2.1) 1→(1.1) 1→(1.1) 1→(1.1) 4→(4.3) 1→(1.1) 82→(87.2)
Symptom duration until initial presentation in the hospital (days)	Mean (range) Median (IQR)		122→7–1095 40→28–89	258.9→42–1095 150→80–312	182→7–1095 80→35–211

*Abbr*.: CLL, chronic lymphatic leukemia; HIV, human immunodeficiency virus; No., number; IQR, interquartile range; TNF‐α, tumor necrosis factor alpha; yr, year

**FIGURE 1:**
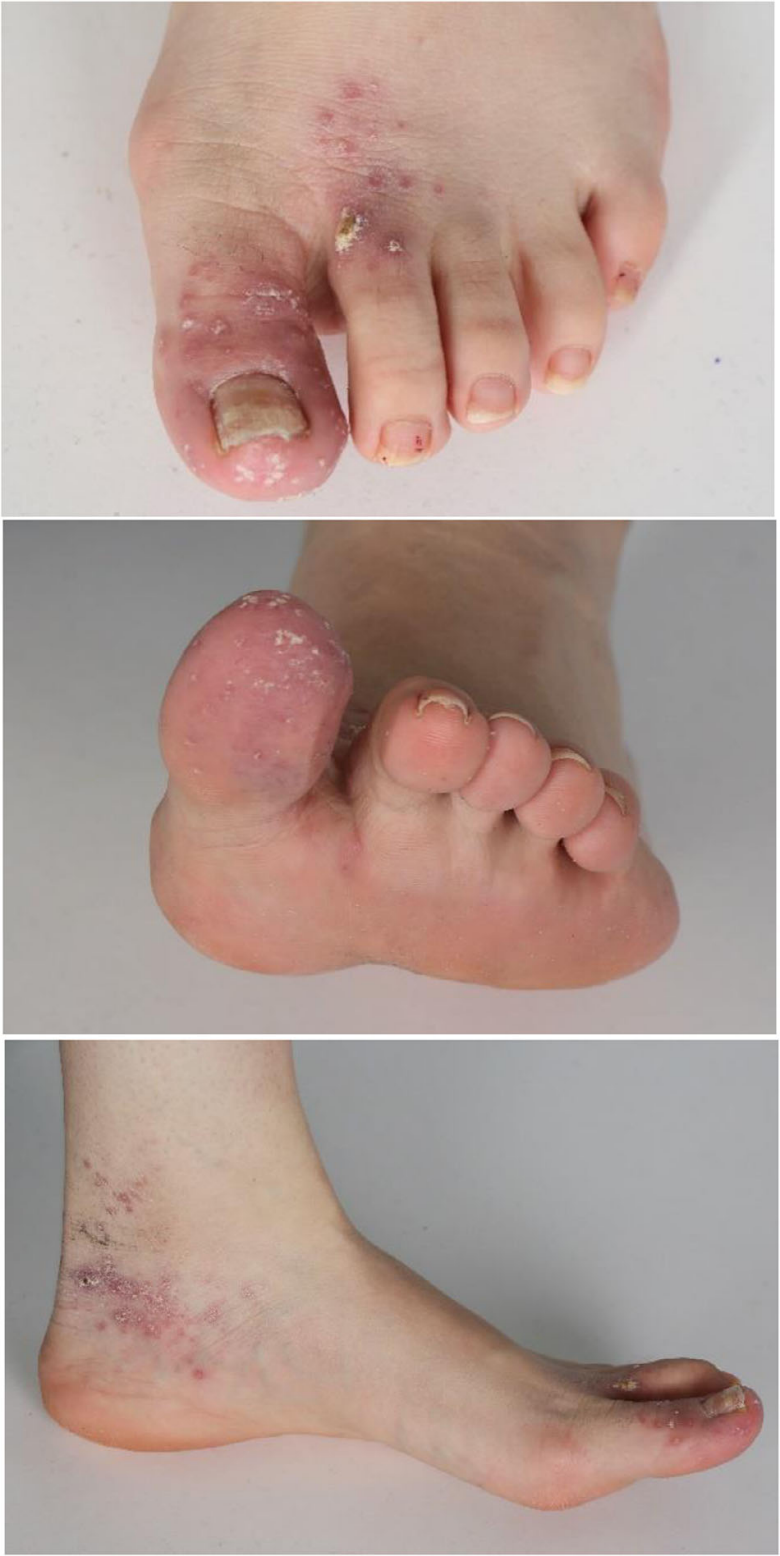
*Mycobacterium marinum* infection of the left foot and toes in a patient with Crohn's disease treated with adalimumab and mesalazine. Multiple grouped granulomatous papules and plaques with crusts on the big toe, dorsal forefoot, and medial ankle.

**FIGURE 2:**
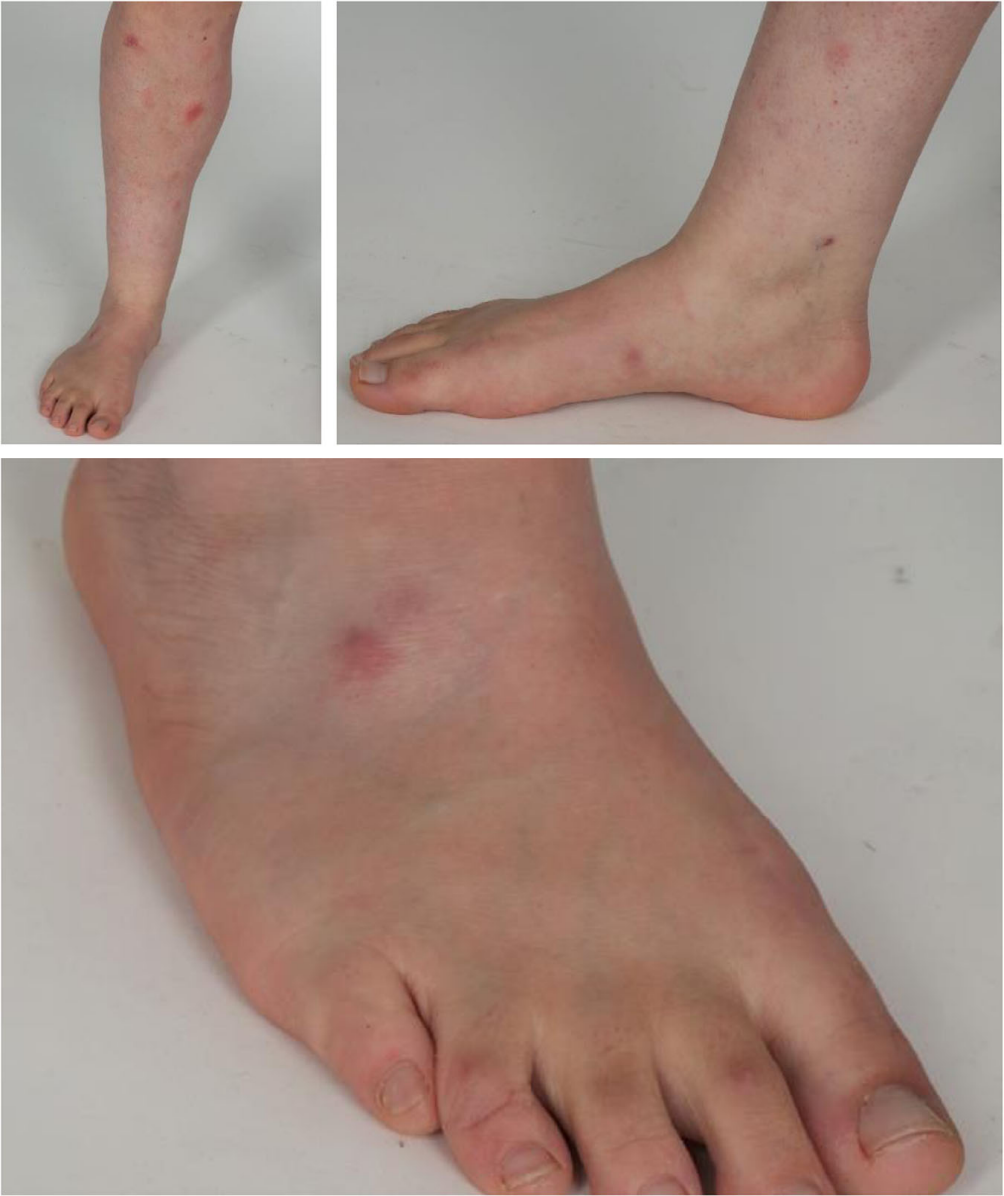
Cutaneous infection with *Mycobacterium* abscessus complex several weeks after liposuction in a patient under immunosuppression with azathioprine. Multiple subcutaneous erythematous nodules and plaques on the right foot and lower leg.

The main infected body site was the upper extremity (89% in cohort‐1 and 62% in cohort‐2) (Table 1). The skin lesions usually presented as solitary or few erythematous papules/nodules (Figure 1, 2), often with erosions/ulcerations or hyperkeratosis, sometimes with adjacent erythema and sporotrichoid distribution. Most patients (90% of cohort‐1, 76% of cohort‐2) were immunocompetent. Treatment with TNF‐receptor‐blockers was prescribed in 4/21 cases in cohort‐2 (19%). The mean symptom duration until the first presentation in the hospital was 122 days (range 7–1,095) in cohort‐1 and 259 (range 42–1,095) in cohort‐2 (Table 1). Before presenting to University Hospitals, 15/73 (21%) cases from cohort‐1 and 7/21 (33%) cases from cohort‐2 were initially misdiagnosed by general practitioners or dermatologists in private practice and had received empirical treatment with streptococcal‐ or staphylococcal‐active antibiotics.

While in all cases of cohort‐2 diagnostics were standardized with biopsies for histology, NAAT and microbial culture each, diagnostic approaches in cohort‐1 were more diverse as expected (Table 2): In 47/73 cases (64.4%), the combination of histology and NAAT and/or culture was performed, while in 14/73 cases (19%) direct pathogen detection by NAAT and/or culture only without histological examination was done. In ten cases of cohort‐1, the only examination performed was histology, and in two cases, the diagnosis was based on clinical symptoms alone.

**TABLE 2:** Diagnostic results and therapeutic regimen.

			Germany (2000–2011)	Cologne (2012–2024)	Combined (2000–2024)
Diagnostics performed – No. (%)	Histology + NAAT + culture Culture + histology Culture + NAAT Histology + NAAT NAAT only Culture only Histology only Clinical symptoms only		21→(28.8) 19→(26.0) 9→(12.3) 7→(9.6) 1→(1.4) 4→(5.5) 10→(13.7) 2→(2.7)	21→(100) 0→(0) 0→(0) 0→(0) 0→(0) 0→(0) 0→(0) 0→(0)	42→(44.7) 20→(21.3) 9→(9.6) 7→(7.4) 1→(1.1) 4→(4.3) 9→(9.6) 2→(2.1)
Pathogen detection with NAAT and/or culture positive – No. (% of cases where either NAAT or culture or both was performed)	Yes No	Only NAAT positive Only culture positive Both positive	47→(77.0) 8→(12.9) 31→(50.8) 8→(12.1) 14→(23.0)	15→(71.4) 6→(28.6) 3→(14.3) 6→(28.6) 6→(28.6)	63→(75.9) 14→(16.9) 35→(42.2) 14→(16.9) 18→(21.7)
Detected pathogens – No. (%)	*M. marinum* *M. chelonae* *M. abscessus* complex^*^ *M. avium* *M. haemophilum* *M. gilvum* *M. vaccae* *M. goodii* Not specified		41→(87.2) 3→(6.3) 0→(0) 1→(2.1) 1→(2.1) 0→(0) 0→(0) 0→(0) 1→(2.1)	10→(66.7) 1→(6.7) 1→(6.7) 0→(0) 0→(0) 1→(6.7) 1→(6.7) 1→(6.7) 0→(0)	52→(82.5) 4→(6.3) 1→(1.6) 1→(1.6) 1→(1.6) 1→(1.6) 1→(1.6) 1→(1.6) 1→(1.6)
Granuloma in histology – No. (%)	Yes No		50→(87.7) 7→(12.3)	17→(81.0) 4→(19.0)	67→(85.9) 11→(14.1)
Time to diagnosis (days)	Mean (range) Median (IQR)		199→3–2,060 90→53–187	276.2→70–1,108 151→118–315	214→3–2,060 98→57–199
Period between suspected diagnosis and confirmed diagnosis in cases with confirmed pathogen detection	Mean (range) Median (IQR)		n/a n/a	37.2→9–90 31→13.5–51.5	n/a n/a
Therapeutic strategy (first‐line)	Antibiotic therapy against NTM only Antibiotic therapy against NTM plus surgical therapy Surgical therapy only No specific therapy		62→(84.9) 7→(9.6) 2→(2.7) 2→(2.7)	19→(90.5) 0→(0) 0→(0) 2→(9.5)	81→(86.2) 7→(7.4) 2→(2.1) 4→(4.3)
Initial antibiotic therapy – No. (% of cases in which antibiotic therapy was initiated)	AB monotherapy AB combination therapy (≥ 2 AB)	Combination of 2 AB Combination of 3 AB Combination of 4 AB	47→(68.1) 22→(31.9) 13→(18.8) 8→(11.6) 1→(1.4)	10→→(52.6) 9→(47.4) 6→(31.6) 2→(10.5) 1→(5.3)	57→(64.8) 31→(35.2) 19→(21.6) 10→(11.4) 2→(2.3)
Initial antibiotics used – No. (% of cases in which AB therapy was initiated)	Clarithromycin Doxycyclin Cotrimoxazole Clarithromycin + rifampicin Minocyclin Rifampicin + ethambutol Ciprofloxacin Doxycyclin + cotrimoxazole Levofloxacin + minocycline Clarithromycin + rifampicin + ethambutol Clarithromycin + doxycycline Levofloxacin Clarithromycin + rifampicin + protionamide Clarithromycin + rifampicin + minocycline Clarithromycin + ethambutol Clarithromycin + ciprofloxacin Clarithromycin + levofloxacin Rifampicin + ethambutol + isoniazid + pyrazinamide		19→(27.5) 13→(18.8) 10→(14.5) 5→(7.2) 5→(7.2) 4→(5.8) 2→(2.9) 2→(2.9) 2→(2.9) 2→(2.9) 0→(0) 1→(1.4) 1→(1.4) 1→(1.4) 1→(1.4) 1→(1.4) 0→(0) 0→(0)	6→(31.6) 2→(10.5) 1→(5.3) 3→(15.8) 1→(5.3) 0→(0) 0→(0) 0→(0) 0→(0) 2→(10.5) 2→(10.5) 0→(0) 0→(0) 0→(0) 0→(0) 0→(0) 1→(5.3) 1→(5.3)	25→(28.4) 15→(17.0) 11→(12.5) 8→(9.1) 6→(6.8) 4→(4.5) 2→(2.3) 2→(2.3) 2→(2.3) 4→(4.5) 2→(2.3) 1→(1.1) 1→(1.1) 1→(1.1) 1→(1.1) 1→(1.1) 1→(1.1) 1→(1.1)
Duration of antibiotic therapy until discontinuation	Mean (range) Median (IQR)		128→10–436 105→77–165	152.3→42–262 140→109.5–199.5	133→10–436 122→81–180
Complications of infection – No. (%)	Yes No	Pain Superinfection Delayed wound healing Cachexia	12→(16.4) 0→(0) 11→(15.1) 1→(1.4) 0→(0) 61→(83.6)	4→(19) 2→(10) 1→(5) 0→(0) 1→(5) 17→(81)	16→(17) 2→(2.1) 12→(12.8) 1→(1.1) 1→(1.1) 78→(83.0)
Adverse events of antibiotic therapy – No. (%)	Yes No	Diarrhea Toxic dermatitis Tendinitis Hearing loss	1→(1.4) 0→(0) 0→(0) 1→(1.4) 0→(0) 72→(98.6)	5→(23.8) 3→(14.3) 1→(4.8) 0→(0) 1→(4.8) 16→(76.2)	6→(6.4) 3→(3.2) 1→(1.1) 1→(1.1) 1→(1.1) 88→(93.6)
Follow‐up visit performed – No. (%)	Yes No (lost to follow‐up)		13→(17.8) 60→(82.2)	15→(71.4) 6→(28.6)	28→(29.8) 66→(70.2)
Recurrence – No. (% of cases with follow‐up visit)	Yes No		2→(15.4) 11→(84.6)	2→(13.3) 13→(86.7)	4→(14.3) 24→(85.7)

*Abbr*.: AB, antibiotic(s); IQR, interquartile range; NAAT, nucleic acid amplification test; No., number

* In this case, NAAT/sequencing was positive for *M. abscessus* complex, but *M. abscessus* and *M. chelonae* were indistinguishable.

Pathogen detection by NAAT and/or culture was successful in the majority of cases in both cohorts (76%) (47/61 cases [77%] in cohort 1 and 15/21 cases [71%] in cohort 2) (Table 2). The most commonly detected pathogen in both cohorts was *M. marinum* (87.2% in cohort‐1, 66.7% in cohort‐2), followed by *M. abscessus/chelonae* (n = 5, with n = 3 *M. chelonae* in cohort‐1 and n = 1 in cohort‐2, and n = 1 *M. abscessus complex*, exact species identification not possible, in cohort‐2). Other pathogens detected were *M. avium*, *M. haemophilum*, in one case of cohort‐1 each and *M. gilvum*, *M. vaccae* and *M. goodii*, in one case of cohort‐2 each (Table 2). Interestingly, in the twelve immunosuppressed patients with positive pathogen detection, the proportion of pathogens other than *M. marinum* was significantly higher (n = 5 *M. abscessus/chelonae* infections, n = 1 *M. haemophilum* infection) than in non‐immunosuppressed patients (without other known risk factors [for example cosmetic procedures]) (p = 0.0004). In six cases with travel history, *M. chelonae* (Thailand), *M. haemophilum* (Kenya, patient living with HIV), *M. gilvum* (Egypt), and *M. marinum* (Israel, Afghanistan, and Slovakia each) were detected.

Antibiotic susceptibility testing was performed in n = 24/40 culture‐positive cases (60%) from cohort‐1 and in 8/9 cases (89%) from cohort‐2 (all *M. marinum*‐positive). Antibiotic resistances were found in 23/24 (96%) of cohort‐1 and in 6/8 cases (75%) of cohort‐2. Detected resistances for *M. marinum* are listed in online supplementary Table S1.

In 67/78 cases of both cohorts (86%), histology showed granuloma formation. In 17/21 (81%) samples with granuloma from cohort‐2 (Table 2), the most common granuloma subtype was messy granuloma in 8/19 (42%), followed by non‐caseating tuberculoid forms in 6/19 (32%) and suppurative granuloma in 5/19 (26%). Ziehl‐Neelsen staining for acid‐fast bacilli was performed in 14/21 (67%) samples and was negative in all cases. In the four cases without granuloma formation, pathological examination revealed inflammatory infiltrates. In three of these four cases, diagnosis was based on positive pathogen detection, and in one case only on clinical symptoms and fish tank exposure.

The mean symptom duration until diagnosis was 199 days (range 3–260) in cohort‐1 and 276 days (range 70–1108) in cohort‐2 (Table 2).

In > 90% of cases, the first‐line therapeutic strategy after diagnosis of cutaneous NTM infection was oral antibiotic therapy (69/73 cases [94.5%] in cohort 1 and 19/21 cases [90.5%] in cohort 2), which was combined with surgery in 7 cases (10%, all in cohort 1). Two patients of cohort‐1 received surgical therapy only, and four patients (two in cohort‐1 and cohort‐2 each) received no specific therapy.

In the majority of cases (47/69 [68.1%] in cohort 1 and 10/19 [52.6%] in cohort 2), antibiotic monotherapy was initiated, most commonly with clarithromycin (19/69 cases in cohort 1 and 6/19 cases in cohort 2), followed by doxycycline or cotrimoxazole (13 and 10 cases in cohort 1 and 2 and 1 case in cohort 2, respectively) (Table 2). The most common combination therapy was clarithromycin + rifampicin in five and three cases of cohort‐1 and cohort‐2, respectively. Other initial antibiotics used are shown in Table 2. In 6/21 cases from cohort‐2, therapy was de‐escalated, most commonly to only clarithromycin or doxycycline (no data available from cohort‐1). Interestingly, in all but one case, patients were treated with antibiotics to which *M. marinum* was not resistant. One patient (cohort‐1) was treated with cotrimoxazole (against which *M. marinum* was tested resistant) in combination with abscess drainage and was completely cured.

The mean duration of antibiotic therapy before discontinuation was 128 days (range 10–436) in cohort‐1 and 152.5 (range 42–262) in cohort‐2 (Table 2). A final visit was more frequently documented in cohort‐2 with n = 15/21 cases (71%) compared to cohort‐1 (n = 13/73, 18%), with recurrences detected in n = 2 cases of each group (see supplementary Table S2 for details).

## DISCUSSION

In the absence of clinical trials, cutaneous NTM infections remain a diagnostic and therapeutic challenge. To date, several retrospective case series of cutaneous NTM infections from different geographical regions have been published. To our knowledge, this is the first larger case series of cutaneous NTM infections in Germany.^25^ Our study provides data on a comparatively large number of 94 patients from two different cohorts diagnosed over a 24‐year‐period. In both cohorts, most cases occurred in immunocompetent men. The main pathogen was *M. marinum* with > 80% of cases, the most common risk factor was aquatic exposure. Furthermore, we demonstrate a long time to diagnosis (median 3 months), a long therapy duration of four months (median), which in most cases was carried out with a single antibiotic. Few complications or side effects of therapy and low recurrence rates were documented.

The pathogen spectrum in our study is comparable to other studies from Europe and the United States, in which *M. marinum* is also the most common pathogen.^11^, ^18^ In contrast, most studies from Asian countries and Australia show mainly RGM‐induced infections.^12^, ^13^, ^26^ Notably, in these studies, preceding surgical and cosmetic procedures, which are associated with RGM‐induced infections,^11^, ^13^, ^14^ were much more common. In our case series, RGM‐induced cutaneous NTM infections were rare and occurred after a surgical procedure (liposuction) only in one case. Of note, this patient was immunosuppressed with azathioprine. Remarkably, all five RGM‐ and one *M. haemophilum*‐induced infection occurred in immunosuppressed patients, which – although the total number of twelve immunosuppressed cases with positive pathogen detection is low – suggests an association of immunosuppression with NTM other than *M. marinum*. However, two of these infections occurred after traveling, which might reflect a different pathogen exposure in these countries. In the future, an increase in RGM‐induced cutaneous NTM infections^11^, ^13^, ^14^ is expectable in Germany, too, also due to cosmetic tourism.^27^, ^28^ Prospective studies with larger patient cohorts analyzing infections depending on risk factors (immunosuppression, travel, surgical or cosmetic procedure, aquatic exposure) will be necessary to further determine the incidence.

As in other case series especially for *M. marinum* infections,^18^, ^29^, ^30^ our study shows a male predominance. In other studies, mainly with RMG‐induced infections after surgical procedures, female patients dominate.^11^, ^12^, ^14^ Whether this is due to a risk factor that is more prevalent in the respective gender (more cosmetic procedures in females,^27^, ^31^ fish tanks – at least in Germany – as a male‐dominated hobby^32^) or whether there is a biological cause for different dominating pathogenic spectra, requires further investigation.

Similar to others,^26^, ^33^ the diagnosis of cutaneous NTM infection in both cohorts was often considered with a significant delay of months after symptom onset and usually first at presentation at the academic institutions. The combination of clinicopathological findings with microbiological analyses and treatment outcomes was necessary to make the correct diagnosis. This is in agreement with other studies.^14^, ^30^ Standard histopathological examination including acid‐fast staining is relatively insensitive in NTM infections. Although most cases in our as well as in previous studies^18^, ^29^, ^33^, ^34^ show granuloma, this can also be caused by other infectious processes such as tuberculosis, leishmaniasis or (subcutaneous) fungal infections. Moreover, acid‐fast bacilli detection rates – 0% in our study – are low^18^, ^30^, ^34^ and – as well as the histological type of granuloma – are not useful in distinguishing between NTM and tuberculous mycobacteria.^19^

Direct pathogen detection remains the gold standard in the diagnosis of NTM infections. Our culture‐positive detection rates of 73.6% in cohort‐1 and 42.8% in cohort‐2 are comparable to other studies.^8^, ^30^ Of note, culture‐positive detection rates depend on optimal cultivation conditions which differ between *M. marinum* and other NTM.^19^ The culture‐positives rates in confirmed *M. marinum*‐cases (the most predominant species in our analyses) were 92.1% for cohort‐1 and 90% for cohort‐2. In best scenarios, a positive culture allows for resistance testing and selection of a suitable therapeutic agent, but it takes several weeks and requires special culture conditions.^19^ Detection by NAAT and/ or sequencing is faster and allows earlier initiation of antimycobacterial treatment. In our study, only 16/38 (42%) and 12/21 (57%) performed NAATs were positive in cohort‐1 and ‐2, respectively. Noteworthy, all five infections with NTM other than *M. marinum* in cohort‐2 were detected only by NAAT and culture was always negative. Some recent studies, in which NAAT was performed as standard,^12^, ^14^, ^18^, ^26^ did not report false‐negative rates of NAAT. A negative NAAT can result from a low density of mycobacteria in the tissue. On the other hand, the identification of NTM by NAAT in human tissue specimens does not necessarily represent an infection since contamination of the samples is frequent.^19^ Of note, NAAT‐based differentiation within the *M. abscessus/chelonae* complex can be challenging.^35^ This can become a problem if there is no positive culture for species differentiation and resistance testing. In contrast to *M. chelonae*, *M. abscessus* can express the *erythromycin resistance methylase* (*erm41)* gene encoding for inducible macrolide resistance,^36^, ^37^ which may affect the choice of the antibiotic agent, since clarithromycin and other macrolides are an important component in the antibiotic therapy of NTM. In our study, this applied to one case – the immunosuppressed patient with infection after liposuction (Figure 2) – in which nucleic acid‐based testing was positive for the M. abscessus/chelonae group, but further differentiation was not possible. After careful consideration and despite the risk of inducible macrolide resistance, we treated the patient with oral clarithromycin + doxycycline for 5 months, resulting in complete healing.

Of note, in cohort‐2, the diagnostic procedure was standardized and always included NAAT, mycobacterial culture, and histopathology from tissue biopsies, while in cohort‐1 diagnostic approaches were more variable. This may be due to the nature of a multiple center assessment in cohort‐1, but also to the earlier time period (NAAT not yet widely available). Given that the sensitivity and specificity of NTM detection by culture or NAAT are limited, and that in some cases only one or the other method was successful in detecting the pathogen, there is a need for a standardized diagnostic procedure‐recommendations for suspected NTM infections: Appropriate material should be obtained in sufficient size from the skin lesion (e.g., three 6 mm punch biopsies) for mycobacterial cultures, NAAT and histopathology. Sources of contamination such as tap water should be avoided and antibiotic use before diagnostics should be limited. The microbiological laboratory should be informed on the suspected diagnosis to cultivate the samples at optimal temperatures (ideally, all specimens should be cultured both at 28 °C to 30 °C and at 35 °C to 37°C to maximize the growth of different NTM species).^19^

In 11/73 (15%) cases of cohort‐1 the final diagnosis was based on clinicopathological features and response to antimycobacterial treatment, an approach that was common in times prior to the wider availability of NAAT and mycobacterial cultures.^30^ It is possible that in these cases, another diagnosis was true, albeit unlikely. The median time to diagnosis of 3 months for all cases mirrors findings from other studies^12^, ^13^, ^14^, ^26^, ^29^ and depended on how the final diagnosis was made: NAAT, clinicopathological assessment only – which is faster – or culture – which often takes several weeks. As in cohort‐2 the diagnostic approach included always biopsies for culture and NAAT, the median time to diagnosis was significantly longer than in cohort‐1 where diagnosis was made without pathogen detection in some cases. In addition to the delay caused by the complex diagnostics, this suggests that cutaneous NTM infections are often only considered once all other alternative diagnoses have been excluded. In our study, 20%–30% of cases were initially misdiagnosed.

The preferred antimicrobial treatment and the spectrum of antibiotics prescribed in both of our cohorts is comparable to other case series. However, in these studies 50%–80% patients underwent additional surgical intervention.^11^, ^12^, ^14^, ^29^, ^33^, ^38^ The observation that antibiotic therapy alone is associated with a higher failure rate in patients with cutaneous NTM infections^7^ could not be confirmed in our study. Both may be partially explained by the dominance of RGM and the inclusion of deeper or invasive infections in other studies.

In most recent case series, therapy was performed with at least two antimicrobial agents,^12^, ^14^, ^29^, ^33^ while others demonstrate good outcomes also after single antibiotic therapy.^18^, ^32^, ^39^, ^40^ The most common antibiotic regimen used in our study was single clarithromycin, followed by single doxycycline or single cotrimoxazole. The most commonly prescribed combination therapy was clarithromycin plus rifampicin, which is in line with the 2007 treatment recommendations for lung infections with *M. marinum*.^20^ Other various combinations and monotherapies were used in single or two cases (Table 2). The variety of antibiotics used in this and in other studies emphasizes the need for standardized regimens. One reason for this is the lack of guidelines based on controlled studies with antibiotics. Furthermore, cutaneous NTM infections are a group of rare skin infections with similar clinical symptoms but several causative pathogens with different biological characteristics (e.g., growth rate, optimum incubation temperature, virulence factors, effective antibiotics) which additionally hampers standardization. Findings on infections with *M. marinum* – the majority of cases found in our study – are therefore not necessarily transferable to other NTM.

Noteworthy, whenever a clinician considers empirical monotherapy with clarithromycin, an infection with a potentially macrolide‐resistant *M. abscessus* should be taken into consideration – particularly in infections following previous surgical interventions. If the risk of complications is low, awaiting species identification and susceptibility testing before initiating treatment is advisable. Our median treatment duration of 4 months mirrors findings from other studies which show median treatment lengths between 2.5–6 months, with a tendency towards longer treatment in RGM‐induced infections.^12^, ^18^, ^26^, ^32^, ^33^

The good outcome with low recurrence rates in our study shows that (single) antibiotic therapy alone (without surgery) can be sufficient to treat cutaneous NTM infections. Clear risk factors for recurrence could not be identified, mainly due to the small number of patients.

Our study has several limitations: All data was collected retrospectively, and the two cohorts differed in their design (cohort‐1 multi‐centric, cohort‐2 single center), and in the period of diagnosis and treatment (cohort‐1 2000‐2012 and cohort‐2 2012‐2024) which must be considered when comparing the data of both groups. In addition to certain strikingly similar aspects between both groups, some major differences (e.g., longer duration of symptoms until first presentation at the clinic, longer duration until correct diagnosis, longer duration of antibiotic therapy, more frequent follow‐up in cohort‐2) become apparent. These differences are most likely center‐related (more standardized history assessment, diagnostics and therapeutic duration in the monocentric cohort‐2) and reflect advances in medical care. Interpretation of the multicentric data from cohort‐1 may be less reliable due to differences in documentation, case recording, diagnostic approaches, and therapy. A more detailed analysis of these aspects is unfortunately not possible due to the lack of detailed data from cohort‐1. In addition, the number of NTM species other than *M. marinum* is too low to draw conclusions on this group of infections, especially with regard to diagnostic and therapeutic recommendations. As we only analyzed cases treated at dermatology departments, patients treated in other departments (e.g., infectious diseases departments) and patients with deeper NTM infections usually treated in orthopedic surgery, were not included.

## CONCLUSIONS

The diagnosis of cutaneous NTM infections is based on the combination of clinico‐histopathologic features, microbiological analyses, and the response to treatment. To increase diagnostic yield, sufficiently large tissue specimens for mycobacterial cultures, NAAT, and histopathological analysis should be obtained before every empirical antibiotic treatment. *M. marinum* is the most common pathogen for cutaneous NTM infection in Germany. There is a need for standardized therapeutic regimens. In our cohort, oral treatment with single clarithromycin or doxycycline for at least 4 months was the most commonly prescribed therapy. The good outcome with low recurrence rates suggests that a single course of clarithromycin or doxycycline can be sufficient, at least in cases where an infection with *M. abscessus* is not suspected.

## CONFLICT OF INTEREST STATEMENT

None.

## Supporting information



Supplementary information
