# Supplementary material for: Cutaneous non‐tuberculous mycobacterial infections: A retrospective study of 94 cases from Germany
Source: J Dtsch Dermatol Ges. 2025 Oct 14;24(5):608–17. doi: 10.1111/ddg.15910 (PMC13140123; doi:10.1111/ddg.15910)
Supplement: Supplementary file 1 — Supplementary information [file DDG-24-608-s001.docx]

|  |  | Germany  (2000-2011) | Cologne  (2012-2024) |
| --- | --- | --- | --- |
| **Resistogram successful** – No. (% of cases with positive culture ) | Yes  No | 24 (60)  16 (40) | 8 (88.9)  1 (11.1) |
| **Antibiotic resistances** – No. (% of all resistances found) | Isoniazid  Pyrazinamid  Rifampicin  Ethambutol  Streptomycin  Trimethoprim-sulfamethoxazole  Levofloxacin  Ciprofloxacin  Moxifloxacin  Doxycyclin  Rifabutin | 21 (34.4)  18 (29.5)  0 (0)  0 (0)  13 (21.3)  1 (1.6)  4 (6.6)  3 (4.9)  0 (0)  0 (0)  0 (0) | 0 (0)  0 (0)  2 (13.3)  3 (20)  0 (0)  3 (20)  0 (0  4 (26.7)  1 (6.7)  1 (6.7)  1 (6.7) |

**Supplemental Table S1. Detected antibiotic resistances in both groups.** Note: Depending on the center, different panels of resistance tests may be used.

| **Patient (P-No., cohort) and risk factors** | **Diagnosis by** | **Prescribed initial therapy** | **Therapy after recurrence and follow-up** |
| --- | --- | --- | --- |
| P53, cohort-1 | Pathogen detection (multiple-resistant *M. marinum*) | Rifampicin plus ethambutol, unknown duration | Lost to follow-up after detection of recurrence |
| P62, cohort-1, immunosuppression with ciclosporine | Pathogen detection (multiple-resistant *M. marinum*) | Doxycyclin only over 135 days | Switch to rifampicin, clarithromycin, and ethambutol over 14 days, followed by clarithromycin only over 70 days, then infection was cleared |
| P08, cohort-2, immunosuppression with etanercept | Compatible clinical symptoms and histology only | Clarithromycin, ethambutol and rifampicin over 4 months, recurrence detected after 2 years | Re-initiation of therapy, still ongoing |
| P11, cohort-2 | Pathogen detection (*M.marinum*) | Clarithromycin over 69 days, recurrence detected after one month | Re-initiation of clarithromycin, then lost to follow-up |

**Supplemental Table S2. Overview of cases with recurrent cutaneous NTM infection and possible explanation of recurrence.**
